# The vibrational and configurational entropy of disordering in Cu_3_Au

**DOI:** 10.1016/j.jallcom.2014.12.215

**Published:** 2015-05-25

**Authors:** Artur Benisek, Edgar Dachs

**Affiliations:** Materialforschung und Physik, Universität Salzburg, Hellbrunnerstr. 34, A-5020 Salzburg, Austria

**Keywords:** Gold, Copper, Alloy, Vibrational entropy, Configurational entropy, Enthalpy

## Abstract

The thermodynamics of disordering in Cu_3_Au have been investigated by measuring the heat capacity of samples with different degrees of long- and short-range order between *T* = 5 and 720 K using relaxation and differential scanning calorimetry. The heat capacities of L1_2_-ordered and fcc-disordered samples show similar behaviour at low temperatures (<300 K). They deviate positively from the linear combination of the end-member heat capacities between ∼30 and 160 K. However, small differences between the two samples exist, as the disordered sample has a larger heat capacity producing a vibrational entropy of disordering of ∼0.05 R. At temperatures higher than 300 K, the heat capacity of the ordered sample shows a prominent lambda-type anomaly at 675 K due to the diffusive L1_2_–fcc phase transition. When starting these measurements with disordered samples, ordering effects are observed between 400 and 620 K, and the disordering reaction is observed at 660 K. Evaluation of the data gives an enthalpy and entropy of disordering at 683 K of 2.0 kJ mol^−1^ and 0.39 R, respectively. However, these values increase with increasing temperature, thereby reducing the short-range order. Because the vibrational and configurational disordering effects become active at different temperature regimes, i.e., the vibrational effects at low temperatures (*T* ≪ 300 K) and the sum of both effects at higher temperatures (*T* > 300 K), they have been successfully separated.

## Highlights

• The heat capacity of disordering (Δ*C_P_*^dis^) was investigated on Cu_3_Au calorimetrically.
• Below 300 K, Δ*C_P_*^dis^ was attributed to vibrational effects.
• Above 300 K, Δ*C_P_*^dis^ was attributed to the sum of vibrational and configurational effects.
• The vibrational and configurational entropy were determined separately as a function of temperature.

## Graphical abstract

**Figure:**
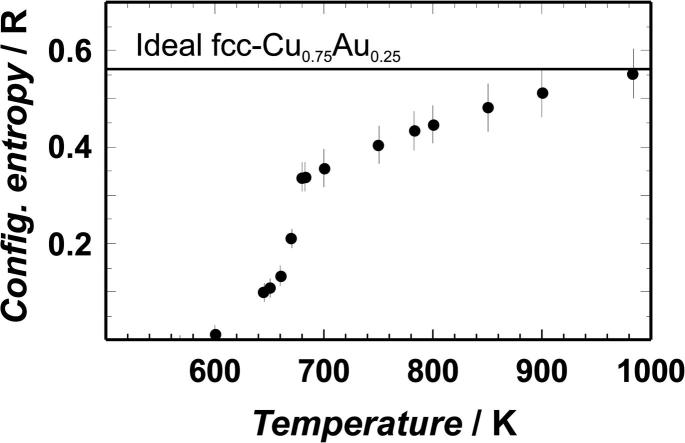

## 1 Introduction

The thermodynamics of Cu_3_Au have been the subject of numerous investigations, e.g., [1–16]. Below ∼580 K, Cu_3_Au has an ordered L1_2_ structure with Au occupying the corners of the unit cell and Cu the face centres [9]. Above 680 K, Cu_3_Au has a face-centered cubic (fcc) structure with a disordered atomic distribution (although there exists short-range ordering). A difference in the vibrational entropy between the ordered and disordered samples (Δ*S*_vib_^dis^) can be observed, which is caused by different heat capacities at temperatures <150 K [1]. Although most studies report that the fcc structure is characterised by a larger vibrational entropy, the results obtained so far are inconsistent (Table 1). Two experimental studies have proposed low values of Δ*S*_vib_^dis^/*R* = 0.02 [4] and 0.03 [1], whereas another experimental study proposed a value of Δ*S*_vib_^dis^/*R* = 0.14 [3]. Theoretical studies have suggested that Δ*S*_vib_^dis^/*R* = 0 [5], 0.08 [7] and 0.07 [8]. The Δ*S*_vib_^dis^ value ranges, thus, from 0 to 0.14 R, which was one reason for the reinvestigation of ordered and disordered Cu_3_Au. In addition to producing data for the vibrational entropy, another aim of this study is to deliver calorimetric results for the configurational entropy. Based on the detailed calorimetric study of Sykes and Jones [11], the energetic behaviour of the atomic rearrangements with increasing temperature can be interpreted as follows: During a heat capacity measurement above 5 K, a fully ordered L1_2_ Cu_3_Au sample does not change its atomic distribution up to 500 K. Its configurational entropy is zero and the vibrational entropy is that of the L1_2_ structure. Between 500 and 670 K, the heat capacity data of such a sample show a lambda-type anomaly due to the L1_2_–fcc phase transition, which changes its atomic distribution. At 670 K, the sample has the vibrational and configurational entropies of the disordered fcc structure. Thus, the lambda peak must contain the entropy difference contributed by both the vibrational and configurational effects due to disordering.

Samples with disordered distributions can be synthesised by quench experiments. They are metastable with respect to their atomic distributions at room temperature and an ordering process takes place, which is, however, very slowly (in ∼2000 days, the difference in heat capacity is reduced to the half [16]). Measuring the heat capacity below 300 K of such disordered samples and comparing these results to data derived from an ordered sample produces a difference in heat capacity that is mainly vibrational in origin [3]. Using these data and those obtained from the disordering reaction, the configurational and vibrational entropy difference due to disordering can be separated. During a heat capacity measurement above 300 K, a quenched sample undergoes first an ordering process followed by a disordering reaction. Using different quench temperatures, the energetic effects of different short-range ordering can be investigated calorimetrically [17].

The heat capacity and the vibrational entropy of a solid solution was often found to deviate from those of the linear combination of the end-members A and B, i.e., they deviate from the Neumann–Kopp rule (for a review, see for example [18,19]). In other words, there exists an excess heat capacity and an excess vibrational entropy of mixing, Δ*S*_vib_^exc^ = *S*_vib_^solid solution^ − (*S_A_* · *X_A_* + *S_B_* · *X_B_*). It is thought that changes in bond length and, consequentially, changes in bond stiffness during compositional variations are responsible for this behaviour (for more details see, e.g., [8,18,20,21]). Using entropy data from the end-members and the measured entropy of Cu_3_Au, the excess entropy of mixing is calculated in this study and compared to that of the most recent thermodynamic data compiled for the Cu–Au system [10]. This approach allows the reliability of the calorimetric results to be tested.

## 2 Experimental methods

### 2.1 Cu_3_Au sample

**Fig. 1:**
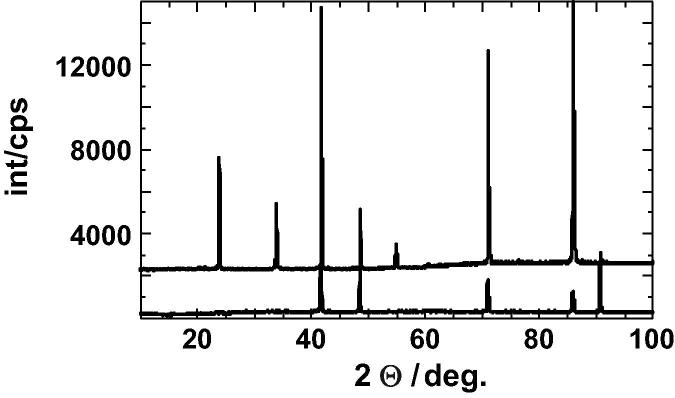
X-ray patterns of fcc disordered and L1_2_ ordered (shifted up by 2000 cps) Cu_3_Au.

Copper and gold powders (purity of >99.9%) were mixed in an agate mortar, pressed to a pellet and melted at 1373 K in an evacuated quartz–glass ampoule, which was used in all following heating experiments. The melted sample was quenched and then pressed to a flat disc and again held at 973 K for 2 days. To produce samples with defined atomic distributions, the samples were equilibrated at different temperatures (for equilibration times see Appendices A and B, that are available as supplementary material from the homepage of this Journal) and quenched into an iced brine bath. The heat capacity measurements started immediately after quenching. The most ordered sample was heated to 783 K then cooled to 658 K where it was held for 1 day followed by further cooling steps: *T* = 628 K for 2 days, *T* = 598 K for 4 days, and *T* = 568 K for 4 days. Finally, the furnace was turned off and slowly cooled down to room temperature. The sample prepared in this way is characterised by strong X-ray superlattice diffraction peaks indicating an ordered Cu–Au distribution. The X-ray patterns of disordered and ordered samples are shown in Fig. 1. The corresponding lattice parameters are *a*_0_/nm = 0.37561 ± 0.00001 and 0.37456 ± 0.00001, respectively. Both values are slightly larger than those of Okamoto et al. [9], who found *a*_0_/nm = 0.375324 and 0.37426 for disordered and ordered samples, respectively. Using their relationship between composition and lattice parameter for disordered Cu_3_Au, a copper mole fraction of *X*_Cu_ = 0.745 is calculated from the observed *a*_0_ value. This composition was verified by electron microscopic investigation, which found that the sample is homogeneous and stoichiometric within the uncertainties.

### 2.2 Relaxation calorimetry (PPMS)

Low-temperature heat capacities from 5 to 300 K were measured using a commercially available relaxation calorimeter (heat capacity option of the PPMS by Quantum Design®). Pieces with ca. 3.3 × 3.3 × 0.3 mm (∼37 mg) were polished and mounted onto the calorimeter platform using Apiezon N grease. The measurements were repeated if the sample coupling, a measure of the quality of the thermal contact between sample and calorimeter platform, was lower than 90% (for details of the relaxation technique, see, e.g., [22,23] and references therein). In such cases, the surface of the Cu_3_Au pieces was reprocessed until a good sample coupling was achieved. The accuracy of the PPMS heat capacities from 100 to 300 K and the entropy at 298.15 K measured on single-crystal and sintered powder samples were found to be better than 0.5% [23].

### 2.3 Differential scanning calorimeter (DSC)

The heat capacity between 300 and 720 K was measured using a power compensated Perkin Elmer Diamond DSC® on samples weighing ca. 165 mg. The DSC measurements were performed under a flow of Ar gas, with the calorimeter block kept at 250 K using a Perkin Elmer Intracooler. Each measurement consisted of a blank run with empty calorimeter chambers and a sample run, where the Cu_3_Au sample was placed into the calorimeter. The heat flow data (difference in heating power between the two chambers) were collected using a temperature scan (heating rate of 5 K/min) and isothermal periods of 3 min before and after the temperature scan. The heat flow versus temperature data from the sample run were shifted and rotated until the data of the isothermal periods agreed with those of the blank run [for details see e.g., 22]. The data from the blank run were then subtracted from those of the sample run to give the net heat flow of the sample. For calculating the heat capacity, the net heat flow data were finally divided by the heating rate and the mass of the sample. The accuracy of the DSC heat capacity data was determined to be better than 0.6% [22].

### 2.4 Evaluation of the raw heat capacity data

In order to calculate the enthalpy and entropy of disordering, the measured heat capacities were integrated numerically using an interpolation function of Mathematica®. The relative uncertainty of the entropy derived from the PPMS heat capacity data amounts to 0.2% for single-crystal and sintered powder samples as determined by a Monte Carlo technique in a previous study [24].

## 3 Results and discussion

### 3.1 Low-temperature heat capacities from 5 to 300 K (PPMS)

**Fig. 2:**
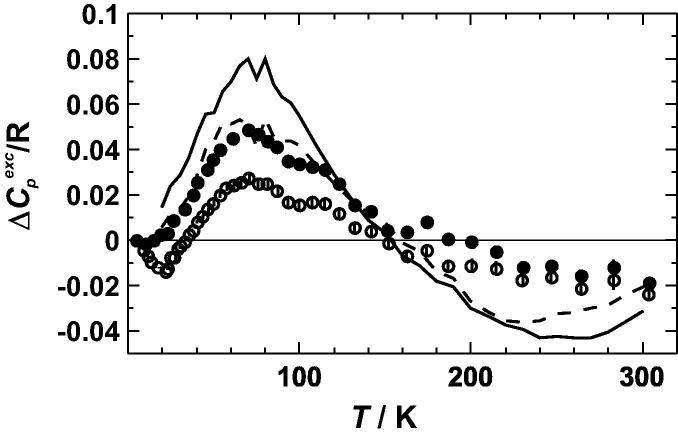
Excess heat capacity of mixing (Δ*C_P_*^exc^) of Cu_0.75_Au_0.25_ as a function of temperature (*T*). The data from the fcc structure are represented by *solid symbols* and a *solid line* whereas those of the L1_2_ structure are represented by *open symbols* and a *broken line*. The *data points* are from this study (error bars represent one standard deviation), and the *lines* are from Yoon and Hultgren [1].

The measured heat capacities in the low temperature range of the different long- and short-range ordered samples are listed in Appendix A (available as electronic supplementary material from the homepage of this journal). The excess heat capacities of mixing (Δ*C_P_*^exc^) were calculated from the measured heat capacities of Cu_3_Au and from the literature values for the heat capacities of pure Cu and Au thoroughly evaluated by Furukawa et al. [25]. The results of the most ordered and disordered samples are plotted against temperature in Fig. 2. The Δ*C_P_*^exc^ of the ordered sample is slightly negative at very low temperature (∼25 K) and positive between 40 and 150 K with a maximum value of 0.025 R at ∼75 K. Above 160 K, it is again slightly negative. The disordered sample shows a Δ*C_P_*^exc^ that is more positive than that of the ordered sample. There is no negative Δ*C_P_*^exc^ at very low temperatures and the Δ*C_P_*^exc^ stays positive from 10 to 200 K with a maximum of 0.05 R at 75 K. Above 200 K, Δ*C_P_*^exc^ is also slightly negative, as is the case with the ordered sample. Fig. 2 compares our excess heat capacity data with those of Yoon and Hultgren [1], which are slightly more positive below 150 K and slightly more negative above 150 K.

**Fig. 3:**
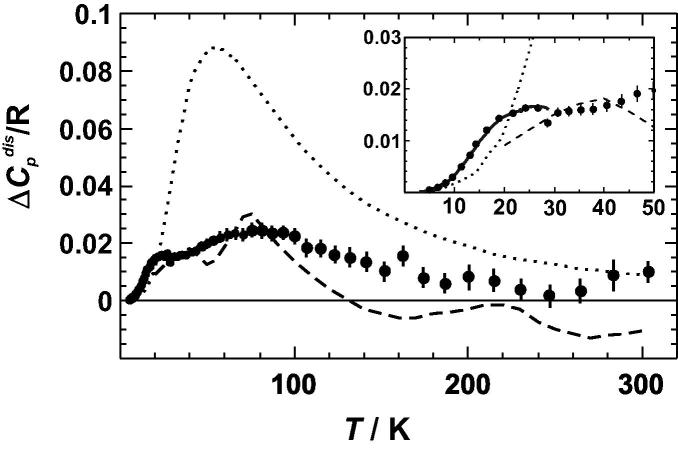
Heat capacity of disordering (Δ*C_P_*^dis^) of Cu_0.75_Au_0.25_ below 300 K. The *data points* are from this study (error bars represent 1 sd), the *solid line* is from Martin [16] with data up to 30 K (inset), the *dashed line* is from Yoon and Hultgren [1], and the *dotted line* is from Nagel et al. [3].

The difference in heat capacity due to disordering (Δ*C_P_*^dis^ = *C_P_*^fcc^ − *C_P_*^L12^) is shown in Fig. 3. Our data show positive Δ*C_P_*^dis^ values with a maximum of 0.025 R at ∼80 K. They are in good agreement with those of [1] but are small compared to [3], whose Δ*C_P_*^dis^ data show a maximum of 0.09 R at 50 K. Below 30 K, the Δ*C_P_*^dis^ values can also be compared with those from the detailed study of Martin [16], showing excellent agreement with our data (inset of Fig. 3).

### 3.2 Heat capacities between 300 and 720 K (DSC)

**Fig. 4:**
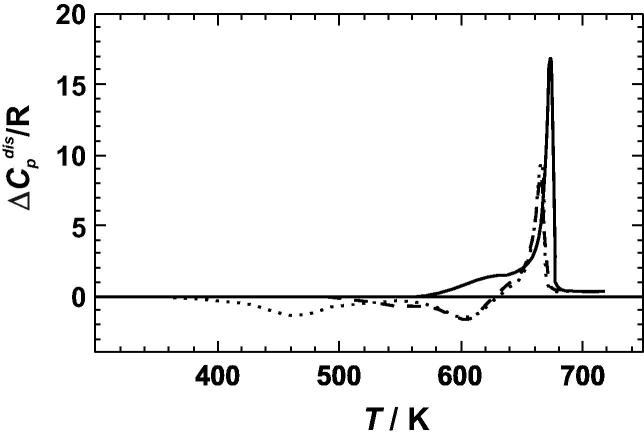
Heat capacity of disordering (Δ*C_P_*^dis^) of Cu_0.75_Au_0.25_ above 300 K calculated via Δ*C_P_*^dis^ = *C_P_*^Cu0.75Au0.25^ − (*C_P_*^Cu^ ∗ 0.75 + *C_P_*^Au^ ∗ 0.25). The end-member heat capacities were taken from [31]. The *solid line* represents the data of the L1_2_ ordered sample, and the *broken* and *dotted lines* represent the data from samples quenched at 703 and 783 K, respectively.

The DSC measured heat capacities of the investigated samples are listed in Appendix B (available as electronic supplementary material from the homepage of this journal). In Fig. 4, the heat capacity difference relative to the linear combination of the end-member heat capacities is plotted. The most ordered sample does not deviate from zero up to 560 K, where the disordering reaction starts. Therefore, it seems plausible that the deviation from zero plotted in Fig. 4 can be solely attributed to the heat capacity of disordering, i.e., the samples do not show any Δ*C_P_*^exc^ at these temperatures. The most ordered sample is characterised by a strong positive (endothermic) peak at 675 K, a temperature slightly higher than the literature value of 664 K [11]. If the DSC measurements were started with disordered samples, the data first show negative deviations and then, at 660 K, a strong positive deviation from the linear combinations of the end-member heat capacities. Such behaviour can be attributed to an ordering of the Cu–Au distribution followed by the pronounced disordering at around 660 K.

Above 680 K, all samples have the same heat capacity, which indicates that they have the same Cu–Au distribution. The samples thus reached near equilibrium conditions during the measurements (heating rate of 5 K/min). Note that the heat capacity is slightly larger than the linear combination of the end-member heat capacities, which is a consequence of further disordering, i.e., the degree of short-range order present at the temperature just above the phase transition is further reduced.

### 3.3 Enthalpy of disordering

**Fig. 5:**
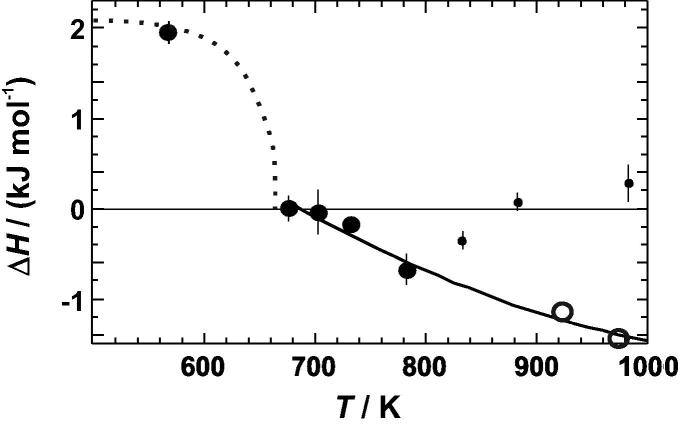
Enthalpy change (Δ*H*) of Cu_0.75_Au_0.25_ plotted against temperature (*T*). The data from this study are represented by *solid symbols*, and data from Kuczyski et al. [12] are represented by *open symbols*. The d*otted line* is taken from Sykes and Jones [11]. The *solid line* represents a fit through the reliable data (see text) above the phase transition.

**Fig. 6:**
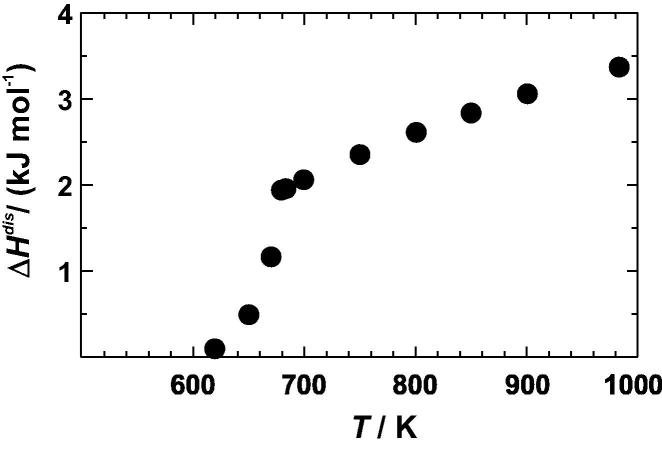
Enthalpy of disordering (Δ*H*^dis^) of Cu_0.75_Au_0.25_ plotted against temperature (*T*).

To calculate the enthalpy change during the DSC experiments, the heat capacity differences of Fig. 4 were integrated from 310 to 680 K. Integration of the calorimetric peak of the most ordered sample (equilibrated at 568 K), resulted in an enthalpy of disordering of 2.0 ± 0.1 kJ mol^−1^ (Table 2). This value is in good agreement with literature values [11] as shown in Fig. 5. The comparison indicates that the sample had an almost fully ordered Cu–Au distribution prior to the measurements. Another sample, which was equilibrated prior to the DSC experiments at temperatures just above the disordering transformation at 703 K, resulted in zero enthalpy of disordering (Table 2), i.e., the area under the negative peaks are equal to that under the positive peak of the heat capacity curves of Fig. 4. The enthalpy change during DSC runs becomes increasingly negative for samples that have been quenched from still higher temperatures of 733 and 783 K (Table 2). This observation is because the samples used in these DSC experiments were more and more disordered at the beginning of the DSC run compared to the atomic distribution at its end. Quench temperatures of more than 800 K, however, resulted in less disordered samples compared to that quenched at 783 K (Table 2). This behaviour is most likely related to an inefficient quench process, which was also found in other studies, [26 and references therein], where potential reasons were discussed. It is also supported by integrating high temperature Δ*C_P_*^dis^ versus *T* data from Kuczynski et al. [12], which produce enthalpy values at e.g., 923 and 973 K that are more negative than the results from our quench experiments at *T* > 800 K. They, however, agree well with the trend of our results from the quench experiments below 800 K using a quadratic fit (Fig. 5). Using the heat capacity differences of the most ordered sample (Fig. 4) and this quadratic fit, the enthalpy of disordering, Δ*H*^dis^, was calculated and plotted in Fig. 6.

### 3.4 Entropy of disordering

**Fig. 7:**
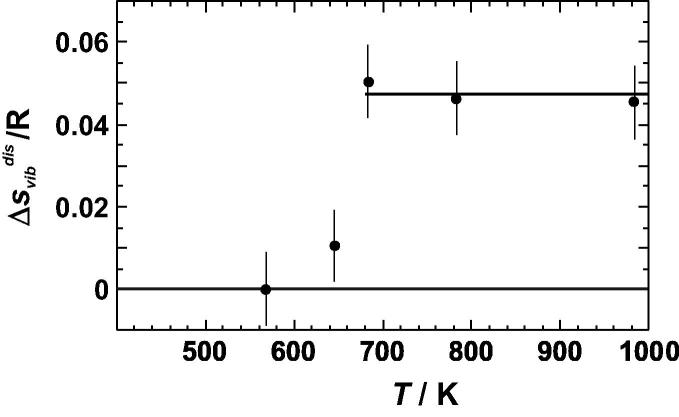
Vibrational entropy of disordering (Δ*S*_vib_^dis^) of Cu_0.75_Au_0.25_ plotted against the quench temperature (*T*).

Let us first consider the vibrational entropy of disordering (Δ*S*_vib_^dis^), i.e., the entropy derived from the integration of Δ*C_P_*^dis^/*T* d*T* between 0 and 300 K (Fig. 3). The corresponding Δ*S*_vib_^dis^ values are plotted against quench temperatures in Fig. 7. If the quench temperature is higher than that of the phase transition (675 K), Δ*S*_vib_^dis^ reaches a value of ∼0.05 R and does not change significantly with a further increase in the quench temperature. Alternatively, Δ*S*_vib_^dis^ was obtained by calculating the entropies at 298.15 K from the *C_P_* data listed in Appendix A, resulting in *S*^vib^/*R* = 4.416 ± 0.009 and 4.463 ± 0.009 for the ordered and disordered (quench temperature of 783 K) samples, respectively. The difference gives a Δ*S*_vib_^dis^ value of 0.046 ± 0.012 R, which agrees with the value obtained by Yoon and Hultgren [1], but it is significantly smaller than that of Nagel et al. [3] (Table 1).

Changes in the vibrational entropy due to disordering were interpreted to be the result of the associated volume changes [27]. The volume (*V*) of the L1_2_ structure is 0.8% smaller than that of the fcc structure (see Section 2.1). Assuming a typical value of 2 for the Grüneisen parameter, *γ* = −*V*/*ω* ∂*ω*/∂*V*, the volume increase of the L1_2_ → fcc phase transition decreases the frequency (*ω*) by 1.6%. Estimating the impact of this change onto the vibrational entropy by using an Einstein heat capacity function leads to an increase in vibrational entropy of 1.6%. This increase corresponds with a Δ*S*_vib_^dis^ value of 0.07 R, which lies near to the measured value.

In a second step, Δ*C_P_*^dis^/*T* of the most ordered sample was integrated stepwise over the temperature interval from 300 to 680 K resulting in increasing entropy of disordering (Δ*S*^dis^) values (Table 3). These values contain both the vibrational and configurational parts of the entropy as introduced in Section 1. To discuss these circumstances from another point of view, let us conduct an analysis of the entropic behaviour of two different Cu_3_Au samples. One sample starts with a fully ordered distribution, which will be denoted “ord”. Its entropy at 0 K (*S*^0^_ord_) is zero. Another sample whose atomic distribution was equilibrated just above the phase transition at 700 K and then quenched to room temperature (denoted “dis”) should have an entropy value at 0 K that is equal to the configurational entropy (*S*_cfg_) of the disordered Cu–Au distribution at 700 K. The entropy difference between both samples at 0 K is given as follows:

(1) $$S_{\text{dis}}^{0}-S_{\text{ord}}^{0}=S_{\text{cfg}}^{700}$$

In the temperature range between 0 and 300 K, the vibrational entropy of disordering becomes effective. The atomic distribution and, consequentially, the configurational entropy are not changed in this temperature range. The difference between the two samples is, therefore, given as follows:

(2) $$S_{\text{dis}}^{0\text{"–"}300}-S_{\text{ord}}^{0\text{"–"}300}=\Delta S_{\text{vib}}^{\text{dis}}$$

Finally, we consider the integral of Δ*C_P_*^dis^/*T* d*T* between 300 and 700 K. For sample “dis”, it is zero because the Cu–Au distribution at the start and at the end of this experiment is the same. This assumption is verified by our measurements, i.e., the Δ*H*^dis^ value of such an experiment was zero (second run of Table 2). Because Δ*H*^dis^/*T* = Δ*S*^dis^, the entropy of disordering in this temperature range for sample “dis” is also zero. For sample “ord”, however, this integral contains the large disordering effect. It is assumed in the literature (e.g., [28]) that it is equal to the configurational entropy. The atomic distribution of sample “ord” is fully ordered at the beginning of the experiment (*S*_cfg_ = 0), and disordered at the end of the experiment with a non-zero *S*_cfg_^700^. The entropy difference between sample “dis” and “ord” in this temperature range can then be written as:

(3) $$S_{\text{dis}}^{300\text{"–"}700}-S_{\text{ord}}^{300\text{"–"}700}=0-S_{\text{ord}}^{300\text{"–"}700}\overset{?}{=}-S_{\text{cfg}}^{700}$$

The entropy difference between the two samples at 700 K is given by the sum of Eqs. (1)–(3). It must be zero at this temperature, because their atomic distributions are the same (see Section 3.2). Thus:

(4) $$S_{\text{dis}}^{700}-S_{\text{ord}}^{700}=S_{\text{cfg}}^{700}+\Delta S_{\text{vib}}^{\text{dis}}-S_{\text{ord}}^{300-700}=0$$

from which it follows that

(5) $$S_{\text{ord}}^{300\text{"–"}700}=S_{\text{cfg}}^{700}+\Delta S_{\text{vib}}^{\text{dis}}\text{.}$$

This means that the integral of Δ*C_P_*^dis^/*T* d*T* between 300 and 700 K of sample “ord” must contain both parts of the entropy, *S*_cfg_^700^ plus Δ*S*_vib_^dis^. The calorimetric peak at 675 K thus contains the configurational and the vibrational entropy of disordering.

**Fig. 8:**
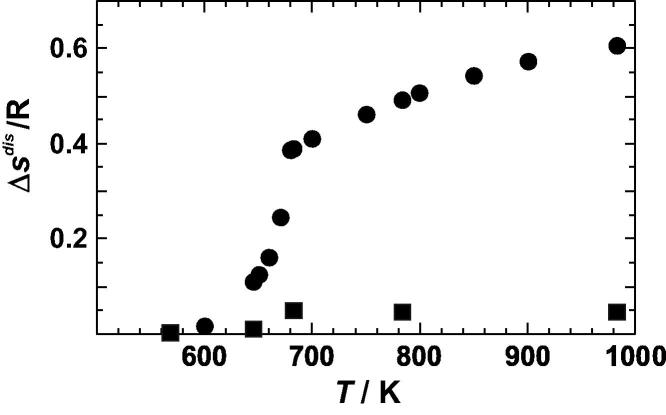
Entropy of disordering (Δ*S*^dis^) of Cu_0.75_Au_0.25_ plotted against temperature (*T*). *Circles* represent the total entropy change (configurational and vibrational). *Squares* represent only the vibrational contributions.

To obtain the Δ*S*^dis^ values at temperatures ⩾700 K, the fitted curve through the Δ*H* versus *T* values (Fig. 5) was differentiated with respect to temperature. It results in Δ*C_P_*^dis^/*R* = 1.408–0.00101 *T*, which is valid between 700 and 1000 K, but do not reflect potential small steps in the disordering process as found by other studies [12,29]. From the so derived Δ*C_P_*^dis^, the entropy of disordering was calculated and is listed in Table 3. Together with the Δ*S*_vib_^dis^ values, the Δ*S*^dis^ values are plotted against temperature in Fig. 8. According to these results, Δ*S*_vib_^dis^ contributes 13% to the whole entropy of disordering at a temperature just above the phase transition. The difference between Δ*S*_vib_^dis^ and Δ*S*^dis^ is the configurational entropy. The corresponding values are listed for some temperatures in Table 4 assuming the most ordered sample has zero configurational entropy. However, from the comparison of its enthalpy of disordering value with the data of Sykes and Jones [11] (Fig. 5), a slightly positive configurational entropy might be estimated (∼0.02 R); however, this value is within the uncertainty limits.

### 3.5 Excess entropy of mixing

According to the most recent thermodynamic data [10] compiled for the Cu–Au system, Cu_0.75_Au_0.25_ is characterised by an almost ideal entropy of mixing at 800 K with only a minor amount of excess entropy (Δ*S*^exc^/*R* = −0.01). Because this value is derived from phase equilibrium data (emf-data [30]), it contains the vibrational and configurational parts of the excess entropy. To derive the excess entropy of mixing from the data from this study, Δ*C_P_*^exc^/*T* of the most ordered sample was integrated over the temperature interval between 0 and 300 K, which yielded ideal vibrational entropy of mixing behaviour for the L1_2_ structure, i.e., Δ*S*_vib_^exc^_L12_/*R* = 0.000 ± 0.009. Between 300 and 800 K, the effect of disordering becomes active, amounting to Δ*S*^dis^/*R* = 0.51 ± 0.04 (Table 3), which is the entropy of mixing value at 800 K. Subtracting the ideal entropy of mixing value, i.e., 0.562 R, from this value results in Δ*S*^exc^/*R* = −0.05 ± 0.04, which agrees with the value of [10]. If the most ordered sample investigated in this study indeed has a non-zero configurational entropy value (i.e., ∼0.02 R) as discussed in Section 3.4, it should be added to Δ*S*^dis^ at 800 K. This approach would improve the agreement of our results with the thermodynamic data set of [10].

## 4 Conclusions

The thermodynamics of disordering in Cu_3_Au have been investigated by calorimetric methods. It could be shown that these methods are able to separate the vibrational and configurational effects. The vibrational entropy of disordering amounts to 13% of the whole entropic disordering effect at a temperature just above the phase transition.

## Figures and Tables

**Table 1:** High temperature limit of vibrational entropy differences in Cu_0.75_Au_0.25_.

	Δ*S*_vib_^exc^/*R* in fcc structure	Δ*S*_vib_^dis^/*R*	Δ*S*_vib_^exc^/*R* in L1_2_ structure
*Experimental studies*			
aFlinn et al. [4]	–	0.02	–
bYoon and Hultgren [1]	0.08	0.03	0.05
cBogdanoff et al. [2]	–	–	0.06
dNagel et al. [3]	–	0.14	–

*Theoretical studies*			
eOzolins et al. [6]	0.12	–	–
Morgan et al. [5]	–	0.0	–
Ozolins et al. [7]	0.18	0.08	0.10
Wu et al. [8]	0.17	0.07	0.10

Δ*S*_vib_^exc^ = *S*_vib_^solid solution^ − (*S*_Cu_ ∗ 0.75 + *S*_Au_ ∗ 0.25). Δ*S*_vib_^dis^ = *S*_vib_^fcc^ − *S*_vib_^L12^.

a Elastic constants of disordered and ordered samples were measured and the Debye temperatures (*Θ*) calculated. The entropy difference was calculated according to *S*^disorder^ − *S*^order^ ≅ 3 k_B_ ln (*Θ*_ord_/*Θ*_dis_).

b Isothermal calorimetry.

c Based on data of inelastic neutron scattering.

d Differential scanning calorimetry above 70 K in addition to calculations using a Born–von Karman model.

e Extrapolated from *X*_Cu_ = 0.5 to 0.75 using a symmetric mixing model.

**Table 2:** Enthalpy change (Δ*H*) during DSC runs for Cu_0.75_Au_0.25_ between the temperatures used to define the atomic configuration prior to the DSC experiment (*T*^prior^) and the end temperature (*T*^end^). Positive values correspond to enthalpy of disordering, whereas negative values correspond to enthalpy of ordering. Numbers in parentheses are the uncertainties and refer to the last digit.

From *T*^prior^ to *T*^end^	Δ*H*/(kJ mol^−1^)
From 568 to 680 K	2.0 (1)
From 703 to 680 K	−0.0 (2)
From 733 to 680 K	−0.17 (8)
From 783 to 680 K	−0.7 (2)
From 833 to 680 K	−0.35 (9)
From 883 to 680 K	0.1 (1)
From 983 to 680 K	0.3 (2)

**Table 3:** Entropy of disordering (Δ*S*^dis^) as a function of temperature (*T*) for Cu_0.75_Au_0.25_. Numbers in parentheses are estimated uncertainties and refer to the last digit.

*T* (K)	Δ*S*^dis^/*R*
600	0.02 (2)
650	0.12 (2)
660	0.16 (2)
670	0.25 (2)
680	0.39 (3)
700	0.41 (4)
750	0.46 (4)
800	0.51 (4)
850	0.54 (5)
900	0.57 (5)

**Table 4:** Vibrational entropy of disordering (Δ*S*_vib_^dis^) and configurational entropy (*S*_cfg_) as a function of temperature (*T*) for Cu_0.75_Au_0.25_. Numbers in parentheses are uncertainties and refer to the last digit.

*T* (K)	Δ*S*_vib_^dis^/*R*	*S*_cfg_/*R*
568	0.000 (9)	0.0 a
645	0.011 (9)	0.10 (2)
683	0.051 (9)	0.34 (3)
783	0.046 (9)	0.45 (4)
983	0.045 (9)	0.56 (5)

Ideal solid solution	0	0.562

a The configurational entropy of the most ordered sample was assumed to be zero. However, when comparing its enthalpy of disordering value with the data of Sykes and Jones [11], a configurational entropy value of ∼0.02 R might be estimated.
