# Supplementary material for: The vibrational and configurational entropy of disordering in Cu3Au
Source: J Alloys Compd. 2015 May 25;632:585–90. doi: 10.1016/j.jallcom.2014.12.215 (PMC4394139; doi:10.1016/j.jallcom.2014.12.215)
Supplement: Supplementary data 1 [file mmc1.doc]

**Appendix A** Measured PPMS heat capacities (*C_P_*) of Cu_0.75_Au_0.25_ of the study

“The vibrational and configurational entropy of disordering in Cu_3_Au” published by Artur Benisek and Edgar Dachs in the

Journal of Alloys and Compounds.

The samples were produced with defined atomic distributions prior to the *C_P_* measurements at different temperatures (*T*^heat^) in heating experiments. The duration (t^heat^) of these experiments are also listed. The uncertainties in *T* and *C_P_* are given as follows:

σ*_T/K_* = +/- *T*/K * (0.001 + 9 * 10^-7^ **T*/K),

σ *_CP/_*_R_ = +/- *C_P_*/R * (0.001 – 1.5 * 10^-6^ **T*/K).

^#^ Temperature and duration of the last cooling step

| *T*^heat^ = 568 K ^#^  t^heat^ = 4 days ^#^ | | *T*^heat^ = 645 K ^#^  t^heat^ = 2 days ^#^ | | *T*^heat^ = 683 K  t^heat^ = 2 days | | *T*^heat^ = 783 K  t^heat^ = 1 day | | *T*^heat^ = 983 K  t^heat^ = 1 day | |
| --- | --- | --- | --- | --- | --- | --- | --- | --- | --- |
| *T*/K | *C_P_/*R | *T*/K | *C_P_/*R | *T*/K | *C_P_/*R | *T*/K | *C_P_/*R | *T*/K | *C_P_/*R |
| | 5.036 | | --- | | 5.401 | | 5.787 | | 6.202 | | 6.659 | | 7.136 | | 7.650 | | 8.199 | | 8.787 | | 9.419 | | 10.10 | | 10.82 | | 11.60 | | 12.44 | | 13.33 | | 14.29 | | 15.32 | | 16.42 | | 17.60 | | 18.87 | | 20.24 | | 21.69 | | 23.24 | | 24.91 | | 26.70 | | 28.62 | | 30.68 | | 32.89 | | 35.26 | | 37.80 | | 40.52 | | 43.44 | | 46.56 | | 49.92 | | 53.51 | | 57.36 | | 61.49 | | 65.91 | | 70.66 | | 75.74 | | 81.17 | | 87.01 | | 93.27 | | 99.97 | | 107.17 | | 114.88 | | 123.14 | | 132.00 | | 141.50 | | 151.68 | | 162.60 | | 174.28 | | 186.84 | | 200.29 | | 214.67 | | 230.09 | | 246.66 | | 264.36 | | 283.34 | | 303.67 | | | 0.0018 | | --- | | 0.0022 | | 0.0026 | | 0.0031 | | 0.0038 | | 0.0046 | | 0.0056 | | 0.0069 | | 0.0085 | | 0.0105 | | 0.0132 | | 0.0166 | | 0.0209 | | 0.0266 | | 0.0337 | | 0.0426 | | 0.0539 | | 0.0678 | | 0.0848 | | 0.1055 | | 0.1304 | | 0.1598 | | 0.1943 | | 0.2348 | | 0.2809 | | 0.3346 | | 0.3915 | | 0.4577 | | 0.5308 | | 0.6112 | | 0.6991 | | 0.7926 | | 0.8925 | | 0.9986 | | 1.109 | | 1.223 | | 1.338 | | 1.455 | | 1.573 | | 1.684 | | 1.797 | | 1.903 | | 2.003 | | 2.104 | | 2.199 | | 2.288 | | 2.366 | | 2.437 | | 2.507 | | 2.567 | | 2.621 | | 2.677 | | 2.720 | | 2.765 | | 2.805 | | 2.837 | | 2.873 | | 2.900 | | 2.931 | | 2.952 | | | 5.036 | | --- | | 5.397 | | 5.809 | | 6.203 | | 6.663 | | 7.136 | | 7.650 | | 8.199 | | 8.787 | | 9.418 | | 10.10 | | 10.82 | | 11.60 | | 12.44 | | 13.33 | | 14.29 | | 15.32 | | 16.42 | | 17.60 | | 18.86 | | 20.23 | | 21.69 | | 23.24 | | 24.91 | | 26.70 | | 28.62 | | 30.68 | | 32.89 | | 35.26 | | 37.80 | | 40.52 | | 43.43 | | 46.56 | | 49.90 | | 53.49 | | 57.36 | | 61.48 | | 65.90 | | 70.65 | | 75.73 | | 81.17 | | 87.00 | | 93.26 | | 99.97 | | 107.16 | | 114.87 | | 123.13 | | 131.99 | | 141.49 | | 151.67 | | 162.57 | | 174.32 | | 186.89 | | 200.32 | | 214.72 | | 230.15 | | 246.70 | | 264.41 | | 283.38 | | 303.72 | | | 0.0019 | | --- | | 0.0023 | | 0.0027 | | 0.0033 | | 0.0040 | | 0.0049 | | 0.0060 | | 0.0073 | | 0.0090 | | 0.0112 | | 0.0140 | | 0.0177 | | 0.0223 | | 0.0281 | | 0.0356 | | 0.0448 | | 0.0565 | | 0.0706 | | 0.0879 | | 0.1088 | | 0.1339 | | 0.1632 | | 0.1979 | | 0.2381 | | 0.2827 | | 0.3367 | | 0.3948 | | 0.4610 | | 0.5342 | | 0.6149 | | 0.7031 | | 0.7967 | | 0.8973 | | 1.004 | | 1.115 | | 1.228 | | 1.343 | | 1.461 | | 1.578 | | 1.691 | | 1.802 | | 1.907 | | 2.006 | | 2.108 | | 2.200 | | 2.289 | | 2.366 | | 2.438 | | 2.506 | | 2.566 | | 2.628 | | 2.679 | | 2.722 | | 2.765 | | 2.807 | | 2.837 | | 2.873 | | 2.900 | | 2.939 | | 2.960 | | | 5.040 | | --- | | 5.409 | | 5.786 | | 6.202 | | 6.661 | | 7.139 | | 7.653 | | 8.201 | | 8.790 | | 9.421 | | 10.10 | | 10.824 | | 11.61 | | 12.44 | | 13.33 | | 14.29 | | 15.32 | | 16.42 | | 17.60 | | 18.86 | | 20.22 | | 21.68 | | 23.24 | | 24.91 | | 26.70 | | 28.62 | | 30.68 | | 32.89 | | 35.25 | | 37.80 | | 40.51 | | 43.43 | | 46.55 | | 49.90 | | 53.50 | | 57.35 | | 61.48 | | 65.90 | | 70.65 | | 75.73 | | 81.16 | | 87.00 | | 93.25 | | 99.94 | | 107.16 | | 114.87 | | 123.13 | | 131.99 | | 141.49 | | 151.67 | | 162.57 | | 174.31 | | 186.87 | | 200.30 | | 214.69 | | 230.11 | | 246.65 | | 264.40 | | 283.37 | | 303.66 | | | 0.0022 | | --- | | 0.0026 | | 0.0032 | | 0.0038 | | 0.0047 | | 0.0058 | | 0.0071 | | 0.0087 | | 0.0108 | | 0.0135 | | 0.0168 | | 0.0211 | | 0.0265 | | 0.0333 | | 0.0417 | | 0.0519 | | 0.0646 | | 0.0797 | | 0.0980 | | 0.1196 | | 0.1452 | | 0.1750 | | 0.2099 | | 0.2502 | | 0.2971 | | 0.3478 | | 0.4068 | | 0.4733 | | 0.5466 | | 0.6272 | | 0.7158 | | 0.8099 | | 0.9112 | | 1.018 | | 1.129 | | 1.245 | | 1.361 | | 1.478 | | 1.596 | | 1.709 | | 1.821 | | 1.927 | | 2.027 | | 2.126 | | 2.217 | | 2.306 | | 2.382 | | 2.452 | | 2.521 | | 2.578 | | 2.641 | | 2.685 | | 2.726 | | 2.773 | | 2.812 | | 2.841 | | 2.875 | | 2.903 | | 2.942 | | 2.964 | | | 5.040 | | --- | | 5.400 | | 5.790 | | 6.208 | | 6.660 | | 7.137 | | 7.650 | | 8.201 | | 8.788 | | 9.421 | | 10.10 | | 10.83 | | 11.61 | | 12.45 | | 13.34 | | 14.30 | | 15.33 | | 16.43 | | 17.61 | | 18.88 | | 20.23 | | 21.69 | | 23.24 | | 24.91 | | 26.70 | | 28.62 | | 30.69 | | 32.89 | | 35.26 | | 37.80 | | 40.52 | | 43.44 | | 46.56 | | 49.91 | | 53.51 | | 57.36 | | 61.49 | | 65.91 | | 70.66 | | 75.73 | | 81.16 | | 87.00 | | 93.25 | | 99.94 | | 107.14 | | 114.85 | | 123.11 | | 131.97 | | 141.46 | | 151.63 | | 162.52 | | 174.30 | | 186.85 | | 200.32 | | 214.72 | | 230.15 | | 246.70 | | 264.41 | | 283.38 | | 303.74 | | | 0.0021 | | --- | | 0.0026 | | 0.0031 | | 0.0038 | | 0.0047 | | 0.0057 | | 0.0070 | | 0.0087 | | 0.0108 | | 0.0135 | | 0.0170 | | 0.0213 | | 0.0267 | | 0.0336 | | 0.0420 | | 0.0523 | | 0.0650 | | 0.0802 | | 0.0984 | | 0.1201 | | 0.1449 | | 0.1749 | | 0.2099 | | 0.2501 | | 0.2976 | | 0.3455 | | 0.4064 | | 0.4726 | | 0.5461 | | 0.6268 | | 0.7165 | | 0.8098 | | 0.9103 | | 1.017 | | 1.128 | | 1.243 | | 1.359 | | 1.476 | | 1.594 | | 1.706 | | 1.815 | | 1.922 | | 2.021 | | 2.122 | | 2.214 | | 2.303 | | 2.379 | | 2.447 | | 2.516 | | 2.573 | | 2.631 | | 2.690 | | 2.732 | | 2.775 | | 2.812 | | 2.843 | | 2.878 | | 2.905 | | 2.937 | | 2.957 | | | | | 5.040 | | --- | | 5.401 | | 5.815 | | 6.208 | | 6.662 | | 7.135 | | 7.647 | | 8.197 | | 8.786 | | 9.414 | | 10.09 | | 10.82 | | 11.60 | | 12.44 | | 13.34 | | 14.30 | | 15.33 | | 16.44 | | 17.62 | | 18.88 | | 20.24 | | 21.70 | | 23.25 | | 24.92 | | 26.72 | | 28.63 | | 30.70 | | 32.90 | | 35.26 | | 37.80 | | 40.52 | | 43.44 | | 46.57 | | 49.92 | | 53.51 | | 57.37 | | 61.50 | | 65.92 | | 70.67 | | 75.74 | | 81.18 | | 87.01 | | 93.26 | | 99.96 | | 107.17 | | 114.89 | | 123.15 | | 132.01 | | 141.50 | | 151.68 | | 162.63 | | 174.32 | | 186.87 | | 200.32 | | 214.71 | | 230.15 | | 246.70 | | 264.41 | | 283.38 | | 303.75 | | | --- | --- | --- | --- | --- | --- | --- | --- | --- | --- | --- | --- | --- | --- | --- | --- | --- | --- | --- | --- | --- | --- | --- | --- | --- | --- | --- | --- | --- | --- | --- | --- | --- | --- | --- | --- | --- | --- | --- | --- | --- | --- | --- | --- | --- | --- | --- | --- | --- | --- | --- | --- | --- | --- | --- | --- | --- | --- | --- | --- | --- | | | --- | --- | --- | --- | --- | --- | --- | --- | --- | --- | --- | --- | --- | --- | --- | --- | --- | --- | --- | --- | --- | --- | --- | --- | --- | --- | --- | --- | --- | --- | --- | --- | --- | --- | --- | --- | --- | --- | --- | --- | --- | --- | --- | --- | --- | --- | --- | --- | --- | --- | --- | --- | --- | --- | --- | --- | --- | --- | --- | --- | --- | --- | | | 0.0022 | | --- | | 0.0026 | | 0.0032 | | 0.0038 | | 0.0047 | | 0.0057 | | 0.0070 | | 0.0087 | | 0.0107 | | 0.0134 | | 0.0168 | | 0.0210 | | 0.0264 | | 0.0332 | | 0.0416 | | 0.0519 | | 0.0646 | | 0.0798 | | 0.0980 | | 0.1197 | | 0.1449 | | 0.1748 | | 0.2098 | | 0.2493 | | 0.2956 | | 0.3505 | | 0.4065 | | 0.4727 | | 0.5462 | | 0.6268 | | 0.7154 | | 0.8096 | | 0.9105 | | 1.017 | | 1.128 | | 1.243 | | 1.358 | | 1.476 | | 1.593 | | 1.706 | | 1.817 | | 1.921 | | 2.020 | | 2.121 | | 2.215 | | 2.304 | | 2.381 | | 2.451 | | 2.520 | | 2.579 | | 2.641 | | 2.685 | | 2.727 | | 2.771 | | 2.810 | | 2.840 | | 2.871 | | 2.901 | | 2.937 | | 2.958 | |
